# Reliability of temporal summation, thermal and pressure pain thresholds in a healthy cohort and musculoskeletal trauma population

**DOI:** 10.1371/journal.pone.0233521

**Published:** 2020-05-29

**Authors:** Nicola Middlebrook, Nicola R. Heneghan, David W. Evans, Alison Rushton, Deborah Falla

**Affiliations:** 1 Centre of Precision Rehabilitation for Spinal Pain, School of Sport, Exercise and Rehabilitation Sciences, College of Life and Environmental Sciences, University of Birmingham, Edgbaston, Birmingham, United Kingdom; 2 NIHR Surgical Reconstruction & Microbiology Research Centre, University of Birmingham, Edgbaston, Birmingham, United Kingdom; Universidad de Leon, SPAIN

## Abstract

Traumatic injuries affect approximately 978 million people worldwide with 56.2 million requiring inpatient care. Quantitative sensory testing (QST) can be useful in predicting outcome following trauma, however the reliability of multiple QST including temporal summation (TS), heat and cold pain thresholds (HPT, CPT) and pressure pain thresholds (PPT) is unknown. We assessed intra (between day) and inter-rater (within day) reliability of QST in asymptomatic participants (n = 21), and inter-rater (within day) reliability in participants presenting with acute musculoskeletal trauma (n = 25). Intra-class correlations with 95% confidence intervals (ICC 3,2), standard error of measurement (SEM) and Bland Altman Plots for limits of agreement were calculated. For asymptomatic participants, reliability was good to excellent for HPT (ICC range 0.76–0.95), moderate to good for PPT (ICC range 0.52–0.93), with one site rated poor (ICC 0.41), and poor to excellent for TS scores (ICC range 0.20–0.91). For musculoskeletal trauma participants reliability was good to excellent for HPT and PPT (ICC range 0.76–0.86), and moderate to good reliability for TS (ICC range 0.69–0.91). SEM for HPT for both sets of participants was ~1°C and an average of 7N for asymptomatic participants and less than 8N for acute musculoskeletal trauma participants for PPT. This study demonstrates moderate to excellent intra and inter-rater reliability for HPT and PPT in asymptomatic participants and good to excellent inter-rater reliability for acute musculoskeletal trauma participants, with TS showing more variability for both sets of participants. This study provides foundations for future work evaluating the sensory function over time following acute musculoskeletal trauma.

## Introduction

Approximately 978 million people worldwide sustain injuries, of which 56.2 million require inpatient care. A high proportion of these injuries are musculoskeletal in nature with 21.7 million sustaining fractures [1]. Acute pain is common, if not expected, following trauma [2] and persistent pain is associated with a poor rate of return to work and reduced activities of daily living [3–5]. Pain assessment in a musculoskeletal trauma population is often limited, with the numerical rating scale (NRS) or visual analogue scale (VAS) most commonly utilised in combination with patient reported questionnaires [6]. Quantitative Sensory Testing (QST), a psychophysical method used to assess sensation and pain perception, can also be used to evaluate the presence of peripheral and central sensitisation; a method commonly used to evaluate pain perception inpatients with spinal cord injuries and musculoskeletal conditions such as low back pain, whiplash and osteoarthritis [7–10]. QST includes multiple tests such as assessing pain thresholds and temporal summation [11]. This can be achieved with different modalities such as thermal or mechanical stimuli [11]. Pain thresholds are defined as the moment that a sensation (e.g. pressure) changes to pain [11], whereas temporal summation can be defined as a gradual increase in the pain response following a number of repetitive stimuli such as heat or pressure [12]. Collectively, cold pain thresholds (CPT), heat pain thresholds (HPT), pressure pain thresholds (PPT) and temporal summation (TS), when used at both local and remote sites of injured tissue, can provide insight into the functioning of A and C fibres together with their central pathways, and can detect the presence of both local (peripheral sensitisation) and more centrally driven symptoms (e.g. secondary hyperalgesia and temporal summation of pain) [11, 13, 14]. QST can therefore be useful to understand which pain mechanisms are at play for an individual patient, knowledge which can inform a more personalised approach to rehabilitation and pain management [14].

Previous studies have found QST to be useful in the assessment of sensory function, and that the results of QST can be used to predict outcome such as the development of chronic pain following injury. For example, secondary hyperalgesia is common in patients with whiplash associated disorders [15–17], and the presence of widespread hyperalgesia including both mechanical and thermal changes at one month following injury can predict poorer outcome at 6 months post injury [18]. QST therefore has the potential to be utilised in a similar manner to predict outcome in a broader musculoskeletal trauma population.

Reliability of QST is well established in asymptomatic populations for PPT, thermal pain thresholds, TS and using the German Research Network on Neuropathic Pain (DNFS) protocol [19–26]. Within symptomatic populations, reliability studies have been conducted in participants with knee osteoarthritis [27], neuropathic pain [28], acute neck pain [29], chronic whiplash [30], spinal cord injury [31, 32] and fractured wrists [33]. However, no reliability study has been performed within an acute hospital environment, evaluating the reliability of QST in people with acute pain following major musculoskeletal trauma. Furthermore, there are limited systematic reviews evaluating the quality of published QST reliability studies. Of those which do exist, they have evaluated thermal sensory testing, conditioned pain modulation and QST test procedures for peripheral joint pain [34–36]. Variable approaches in reporting, methods used and statistical analysis were highlighted in these reviews with a call for higher quality reliability studies to be conducted. There is the need for a rigorous reliability study of multiple QST measures within an asymptomatic population and the need to establish the reliability of these measures for people with acute pain following musculoskeletal trauma.

The aims of this study were to 1) establish both intra and inter-rater reliability of HPT, CPT, PPT and TS measures in healthy asymptomatic volunteers and 2) establish inter-rater reliability of the same measures for people with acute pain following musculoskeletal trauma whilst based in an acute hospital setting.

## Materials and methods

Ethical approval was obtained from the University of Birmingham Ethics Committee (ERN_17–0893) for asymptomatic participants and NHS ethical approval, HRA approval and individual site confirmation (17/WA/0421/IRAS 229790) was obtained for acute musculoskeletal trauma participants. The study was conducted according to the Declaration of Helsinki. All data are available at DOI: 10.6084/m9.figshare.12102495.

### Study design

Two test-retest reliability studies were conducted. A within (inter-rater) and between day (intra-rater) reliability study was conducted for asymptomatic participants, and within (inter-rater) day reliability study was conducted for participants with acute musculoskeletal trauma. For both intra and inter-rater reliability all measures of HPT, CPT, PPT and TS were evaluated. For reliability studies using 2 raters, a sample size of n≥19 was required, based on a power calculation of 5% significance with true reliability exceeding 0.7 [37, 38]. Three raters were recruited with varying levels of QST experience (range 6 months to 10 years). Rater 1 and 2 completed the asymptomatic intra and inter-rater study and rater 1 and 3 completed the acute musculoskeletal trauma inter-rater study. Raters underwent training prior to data collection in the use of the equipment and testing procedure. This included training on the testing procedure of all modalities to ensure the same wording was used by each rater to the participants, as well as training on the use of equipment to ensure that a similar technique was used for all raters. For both sets of participants, room temperature and background noise level were not strictly controlled.

### Participants

#### Asymptomatic participants

Participants were recruited from the staff and student population of the University of Birmingham, United Kingdom via poster advertisement. All willing participants were screened to ensure they met the inclusion criteria, and written consent was gained if eligible. Inclusion criteria were adults (aged ≥18) with the capacity to understand both written and verbal English language. Exclusion criteria included any pain which had affected activities of daily living within the last month, or neurological and rheumatological conditions (e.g. Parkinson’s disease, multiple sclerosis, rheumatoid arthritis etc) [39].

#### Acute musculoskeletal trauma participants

Participants were recruited from a major trauma centre in the United Kingdom. All potential participants were screened by a team of research nurses. If eligible, participants were approached by one of the research nurses and were given a participant information sheet. All participants who were interested in the study were then approached by one of the research team to obtain consent. Musculoskeletal trauma was defined as any trauma (e.g. from a road traffic accident) which involved the musculoskeletal system. This included fractures, stab and gunshot wounds. The broad definition is in keeping with studies and systematic reviews evaluating musculoskeletal trauma [40–42]. Inclusion criteria included being admitted to the major trauma centre within 14 days of injury, adult (aged ≥16), and the capacity to understand both written and verbal English language. The difference in inclusion criteria age between the two sets of participants was due the University population where the asymptomatic participants were recruited were aged ≥18, whereas within the hospital setting the adult age was defined as ≥16 [40]. Therefore, to ensure we recruited all potential eligible participants the age range was kept at ≥16 for the acute musculoskeletal trauma participants. Exclusion criteria included an acute intra-cranial lesion with a Glasgow Coma Scale score of ≤14, any neurological or rheumatological disorders, ongoing terminal illness (e.g. comorbid cancer) with short life expectancy or prolonged use of corticosteroids [40]. The inclusion and exclusion criteria for the acute musculoskeletal trauma participants is intentionally diverse in order to evaluate reliability of these measures within this environment and population. By evaluating reliability in a lab-based environment on asymptomatic participants and then within a real-life environment allows applicability to a real-life clinical situation where these measures can be used.

### Equipment

For both sets of participants the same equipment was used. For the thermal testing (HPT, CPT), the TSA-II NeuroSensory Analyzer thermal stimulator (Medoc Ltd, Israel) and accompanying software with a 30 x 30mm peltier thermode was used. For both PPT and TS testing, a handheld digital pressure algometer (Series 7 force gauge, Mark-10 corporation, USA) with custom made software (LabVIEW, National Instruments, USA) was used.

### Procedure

#### Asymptomatic intra and inter-rater study

A total of four sessions took place over two days. Two sessions took place on each day with a minimum of two hours between sessions to allow a washout period to take place and avoid potential learning effects such as participants remembering the pain ratings of previous sessions for TS [37]. There was a minimum of 48 hours and a maximum of seven days between the two testing days. Six sites in total were tested in each participant including upper limb (extensor carpi radialis muscle belly), lower limb (tibialis anterior muscle belly) and spinal (lumbar erector spinae); all tested bilaterally. A combination of upper and lower limb and spinal sites were chosen to test within the asymptomatic population in an attempt to reflect the range of potential sites which maybe encountered for the acute musculoskeletal trauma participants [5]. Each site was marked with a Schuco Surgical Skin Marking Pen at the first session to allow the same sites to be tested in each session. The same six sites were used at each session for every participant.

#### Acute musculoskeletal trauma inter-rater study

A total of two sessions on the same day with a minimum of two hours between sessions to allow a washout period to take place and avoid potential learning effects was observed [37]. Due to the changeable nature of symptoms following musculoskeletal trauma, together with known sensory changes i.e. peripheral sensitisation following injury to tissue/inflammation, [43], intra-rater reliability testing on a different day was deemed unsuitable due to the likelihood of capturing change of symptoms and sensory changes over time rather than assessing reliability of testers. Due to patient burden, it was deemed unnecessary to test six sites as per the asymptomatic participants. Additionally, these sites would not have been applicable to the injuries in which the participants sustained. Therefore a total of two sites were tested for each participant–a local site within or close to the same dermatome of the injury and a remote site which was on the opposite side to the injury where possible. The choice of both the local and remote site depended on injuries the participant had sustained resulting from trauma and therefore could not be standardised across all participants [40]. Each site was marked with a Schuco Surgical Skin Marking Pen at the first session, and the same sites were used for both sessions but were individual to each participant.

#### Testing procedure

The testing procedure for both sets of participants was standardised. For both HPT, CPT and PPT, a method of limits design [44] was used. For each modality, two measurements were taken per session and the mean calculated. A minimum of 30 seconds was observed between each measurement. Within the first session a period of familiarisation was conducted prior to testing before each modality [40].

#### Thermal testing

Thermal testing was tested using skin contact stimulation. For HPT and CPT temperature either increased or decreased from a baseline of 32°C at a rate of 1°C/second until the maximum temperature of 50.5°C for HPT and 0°C for CPT had been reached in which the temperature then automatically returned to baseline. The participants were instructed to press a button when the sensation changes from a warm/cold sensation to one of pain. Once the button was pressed, the temperature returned to baseline at the same rate of 1°C/Second [44].

#### Pressure pain threshold and temporal summation testing

For the PPT testing, the tip of the force gauge was applied perpendicular to the skin prior to testing and then pressure was applied at a constant rate of 5 newtons/second [19, 44, 45]. The participant was instructed to press a button when the pressure sensation changed to pain. Upon pressing the button, the pressure was released immediately.

For TS testing, the mean PPT score from that session was used. Ten consecutive pulses were applied at this threshold level. The pressure was increased over five seconds, held for one second before being released immediately, with a one second interval before the next pulse [46, 47]. At each peak pulse, the participant was asked to rate their pain on an NRS of 0–10 with 0 being no pain and 10 being pain as worse as it could be.

### Order of testing

To minimise learning effects and potential bias, the order of modalities, site and raters were randomised. Due to the nature of TS testing, and the requirement of session PPT scores, this was always the last test performed. For consistency, one rater performed all of the site markings for each participant prior to testing commencing.

### Data analysis

For measures of reliability, intraclass correlation coefficients, two-way mixed effects, absolute agreement (ICC 3,2) for PPT, CPT and HPT and ICC [3,10] for TS, with 95% confidence intervals (CIs) were calculated. The ICC model was chosen *a priori* with a two-way mixed effects model (model 3) chosen as the raters in the study were only raters of interest, and an average measurement as a mean of more than one measurement was calculated [48]. Bland Altman Plots were calculated for limits of agreement (LOA) as well as standard error of measurement (SEM) calculated. For interpretation of the Bland Altman Plots in terms of good agreement, the majority of the points were required to be within the 95% limits of agreement, with an even distribution of points on both sides of the mean difference to indicate no systematic bias [49]. The descriptive data for CPT, HPT and PPT are reported as the mean of two measurements (SD). CPT and HPT are reported in degrees Celsius, and PPT in Newtons. For TS analyses and to capture the variation in the pain ratings over the 10 pulses, the mean NRS of pulses 1 to 4 (M1), 5 to 7 (M2), and 8 to 10 (M3) was calculated as well as the ratio between M3 and M1 [20]. In order to calculate the ratio where a number greater than 0 was required, 0.1 was added to all temporal summation scores. SPSS (version 25) was used for analyses. For interpretation of ICC results the following criteria were used: <0.5 = poor, 0.5–0.74 = moderate, 0.75–0.9 = Good and >0.91 = Excellent [50, 51].

## Results

### Participant demographics

#### Asymptomatic participants

A total of 21 participants were recruited. One participant was excluded from data analyses due to extra-ordinarily high ratings on all measures, the majority of which reached the safety limits of the testing equipment. The sample size was reduced to 20 participants (50% male) for analysis. Mean age was 27.55 (Standard Deviation (SD) 8.06).

#### Acute musculoskeletal trauma participants

A total of 25 participants were recruited. Five participants were excluded from data analysis for the following reasons: slow reaction times with an inability to press the button when pain thresholds were reached (n = 2), second data set not collected due to reduction in mental capacity (n = 1), or discharged from hospital before a full data set had been collected (n = 2). The mean age of participants (70% male) included in data analyses was 44.8 (SD 19.32). Participant demographics are summarised in Table 1 with injury characteristics for the acute musculoskeletal trauma participants summarised in Table 2.

**Table 1:** Patient characteristics for both asymptomatic and musculoskeletal trauma participants.

	Asymptomatic	Acute Musculoskeletal Trauma
Age, mean (SD)	27.55 (8.06)	44.8 (19.32)
Sex, male n (%)	10 (50%)	14 (70%)
Height, cm (SD)	172.72 (9.50)	170.4 (12.96)
Weight, kg (SD)	72.89 (15.62)	77.45 (14.67)
Body Mass Index (SD)	24.32 (4.71)	26.61 (3.99)

**Table 2:** Injury characteristics for acute musculoskeletal participants.

Number of Injuries	Single Injury	10
	Multiple Injury	10
Location of Injury	Upper Extremity	1
	Lower Extremity	16
	Trunk	1
	Upper and Lower Extremity	2
Type of Injury	Fractures	19
	Soft Tissue	1

### Intra-class correlation coefficients and standard error of measurement

#### Asymptomatic participants

*Heat pain thresholds*. Intra-rater reliability at all sites was good or excellent (ICC range 0.76, 0.91). Inter-rater reliability for both days of testing at all sites was good or excellent (ICC range 0.83, 0.95). The average SEM for rater 1 across all sites was 0.97°C for day 1 and 0.84°C for day 2. For rater 2, the average SEM was similar at 0.89°C for day 1 and 0.81°C for day 2. All ICCs with 95% CIs, SEM and means (SD) for all sites for HPT are reported in Table 3.

**Table 3:** Intraclass correlations, means and standard error of measurement for heat pain thresholds for the asymptomatic participants.

Site	ICC (95% CI) Intra-Rater		ICC (95% CI) Inter-Rater		SEM Day 1 (^0^C)		SEM Day 2 (^0^C)		Mean (SD) Day 1 (^0^C)		Mean (SD) Day 2 (^0^C)	
	Rater 1	Rater 2	Day 1	Day 2	Rater 1	Rater 2	Rater 1	Rater 2	Rater 1	Rater 2	Rater 1	Rater 2
ULR	0.76 (0.41, 0.90)	0.88 (0.71, 0.95)	0.88 (0.70, 0.95)	0.94 (0.85, 0.98)	1.13	1.10	0.76	0.74	44.75 (3.27)	45.19 (3.18)	45.70 (3.08)	45.48 (3.01)
ULL	0.82 (0.56, 0.93)	0.91 (0.77, 0.97)	0.90 (0.74, 0.96)	0.91 (0.76, 0.96)	1.34	1.04	0.99	1.09	44.10 (4.18)	44.58 (3.23)	45.00 (3.20)	45.35 (3.54)
LLR	0.78 (0.43, 0.91)	0.85 (0.42, 0.95)	0.95 (0.88, 0.98)	0.88 (0.61, 0.96)	0.59	0.54	0.94	0.72	44.97 (2.67)	44.97 (2.43)	45.18 (2.67)	46.05 (2.05)
LLL	0.76 (0.41, 0.91)	0.90 (0.74, 0.96)	0.83 (0.57, 0.93)	0.94 (0.86, 0.98)	0.97	0.95	0.52	0.59	45.00 (2.32)	45.51 (2.28)	45.84 (2.19)	45.71 (2.47)
SR	0.83 (0.57, 0.93)	0.91 (0.78, 0.97)	0.92 (0.81, 0.97)	0.91 (0.76, 0.96)	0.71	0.67	0.89	0.88	43.13 (2.54)	43.51 (2.41)	44.03 (2.92)	43.37 (2.90)
SL	0.77 (0.42, 0.91)	0.94 (0.85, 0.98)	0.84 (0.61, 0.94)	0.92 (0.80, 0.97)	1.07	1.04	0.91	0.83	42.96 (2.70)	43.35 (2.63)	44.07 (3.20)	43.63 (2.93)

ULR, Upper Limb Right; ULL, Upper Limb Left; LLR, Lower Limb Right; LLL, Lower Limb Left; SR, Spine Right; SL, Spine Left; ICC, Intraclass correlation; CI; Confidence Interval; SD, Standard Deviation; SEM, Standard Error of Measurement

*Cold pain thresholds*. Ten participants were excluded from cold data analyses as pain was not evoked within the safety limit of the thermal testing equipment in three or more sessions for each site. Of the remaining 10 participants, only two participants had a full data set for all sessions and sites. Eight participants had a partial data set where either intra or inter-rater reliability could be calculated. Due to low number of participants included in data analysis, it was deemed insufficient to perform ICCs and SEM, therefore descriptive data only is reported. A summary of means and SDs can be found in Table 4.

**Table 4:** Asymptomatic participant’s descriptive data results for cold pain threshold.

Site	Means (SD) (^0^C) Intra-Rater		Site	Means (SD) (^0^C) Inter-Rater		Site	Mean (SD) all participants Day 1 (^0^C)		Mean (SD) all participants Day 2 (^0^C)	
	Rater 1	Rater 2		Day 1	Day 2		Rater 1	Rater 2	Rater 1	Rater 2
ULR	N = 7	N = 6	ULR	N = 9	N = 7	ULR	N = 9	N = 9	N = 8	N = 7
Session 1	18.55 (10.71)	18.06 (9.09)	Rater 1	15.49 (11.10)	16.50 (8.52)		15.49 (11.10)	14.16 (9.44)	14.81 (9.23)	14.23 (8.95)
Session 2	6.03 (9.26)	15.60 (8.97)	Rater 2	14.16 (9.44)	14.23 (8.95)					
ULL	N = 8	N = 6	ULL	N = 8	N = 6	ULL	N = 9	N = 8	N = 8	N = 6
Session 1	18.87 (10.50)	20.43 (8.54)	Rater 1	19.24 (9.93)	20.60 (7.77)		17.54 (10.59)	16.81 (9.85)	16.30 (10.34)	20.23 (8.76)
Session 2	16.30 (10.34)	20.23 (8.76)	Rater 2	16.81 (9.85)	20.23 (8.76)					
LLR	N = 4	N = 4	LLR	N = 5	N = 4	LLR	N = 6	N = 5	N = 5	N = 5
Session 1	21.53 (7.45)	23.23 (1.97)	Rater 1	20.19 (9.57)	18.53 (10.79)		18.68 (9.32)	19.47 (8.58)	15.03 (12.18)	18.71 (9.44)
Session 2	18.04 (11.73)	22.78 (2.90)	Rater 2	19.46 (8.58)	17.78 (10.63)					
LLL	N = 7	N = 6	LLL	N = 7	N = 6	LLL	N = 7	N = 7	N = 7	N = 6
Session1	17.71 (7.89)	17.90 (7.35)	Rater 1	17.71 (7.89)	18.16 (6.44)		17.71 (7.89)	15.55 (9.14)	16.14 (7.94)	18.75 (6.18)
Session 2	16.14 (7.94)	18.75 (6.18)	Rater 2	15.55 (9.14)	18.75 (6.18)					
SR	N = 7	N = 6	SR	N = 6	N = 6	SR	N = 7	N = 6	N = 7	N = 6
Session 1	22.78 (8.44)	23.40 (4.76)	Rater 1	25.62 (4.20)	21.65 (6.75)		22.78 (8.44)	23.40 (4.76)	19.78 (7.90)	20.50 (6.71)
Session 2	19.78 (7.90)	20.50 (6.71)	Rater 2	23.40 (4.76)	20.50 (6.71)					
SL	N = 6	N = 5	SL	N = 6	N = 5	SL	N = 6	N = 6	N = 6	N = 5
Session 1	22.48 (6.92)	20.67 (9.08)	Rater 1	22.48 (6.92)	19.45 (7.35)		22. 48 (6.92)	19.83 (8.38)	17.36 (8.33)	18.06 (8.51)
Session 2	17.36 (8.33)	18.06 (8.51)	Rater 2	19.83 (8.38)	18.06 (8.51)					

ULR, Upper Limb Right; ULL, Upper Limb Left; LLR, Lower Limb Right; LLL, Lower Limb Left; SR, Spine Right; SL, Spine Left; N, number of participants; SD, Standard Deviation

*Pressure pain thresholds*. For intra-rater reliability, rater 1 demonstrated a wide range of ICC scores which ranged from poor to good reliability (ICC range 0.41, 0.83), with the lower limb sites and one upper limb site demonstrating lower ICCs compared to spinal sites. Rater 2 demonstrated higher intra-rater reliability (ICC range 0.70, 0.88), although demonstrated wide CIs. Inter-rater reliability for day 1 of testing was rated as moderate to excellent (ICC range 0.66, 0.92) depending on site, with day 2 demonstrated improved inter-rater reliability rated as good to excellent (ICC range 0.80, 0.93). The average SEM across all sites for rater 1 was 7.12 newtons (N) for day one and 5.96 for day 2. For rater 2, the average SEM was slightly lower at 6.93N for day one and 5.98N for day two. ICCs with 95% CIs, SEM and means for all sites for PPT are reported in Table 5.

**Table 5:** Intraclass correlations, means and standard error of measurement for pressure pain thresholds for the asymptomatic participants.

Site	ICC (95% CI) Intra-Rater		ICC (95% CI) Inter-Rater		SEM Day 1 (N)		SEM Day 2 (N)		Mean (SD) Day 1 (N)		Mean (SD) Day 2 (N)	
	Rater 1	Rater 2	Day 1	Day 2	Rater 1	Rater 2	Rater 1	Rater 2	Rater 1	Rater 2	Rater 1	Rater 2
ULR	0.78 (0.44, 0.91)	0.75 (0.35, 0.90)	0.66 (0.15, 0.86)	0.93 (0.83, 0.97)	7.14	6.64	3.77	4.15	33.70 (12.17)	36.69 (11.32)	38.62 (14.68)	37.80 (16.17)
ULL	0.65 (0.11, 0.86)	0.70 (0.27, 0.88)	0.66 (0.14, 0.87)	0.80 (0.49, 0.92)	7.35	4.20	6.45	5.65	33.07 (12.61)	31.30 (7.20)	35.53 (14.35)	33.71 (12.57)
LLR	0.41 (-0.43, 0.77)	0.77 (0.43, 0.91)	0.79 (0.48, 0.92)	0.81 (0.51, 0.92)	9.91	9.11	9.00	7.82	44.97 (21.53)	50.77 (19.78)	51.80 (20.48)	54.13 (17.80)
LLL	0.52 (-0.11, 0.80)	0.88 (0.68, 0.95)	0.75 (0.38, 0.90)	0.90 (0.75, 0.96)	7.11	9.14	5.26	5.56	43.64 (14.17)	49.49 (18.22)	50.59 (16.80)	50.29 (17.76)
SR	0.83 (0.57, 0.93)	0.86 (0.64, 0.94)	0.92 (0.77, 0.97)	0.92 (0.74, 0.97)	6.03	6.77	5.42	6.87	48.11 (21.32)	54.12 (23.93)	54.33 (19.04)	60.72 (24.14)
SL	0.76 (0.41, 0.91)	0.87 (0.32, 0.96)*	0.90 (0.76, 0.96)	0.93 (0.74, 0.98)*	5.15	5.71	5.87	5.80	48.62 (16.63)	51.67 (18.42)	55.72 (23.25)	60.92 (22.57)

ULR, Upper Limb Right; ULL, Upper Limb Left; LLR, Lower Limb Right; LLL, Lower Limb Left; SR, Spine Right; SL, Spine Left; ICC, Intraclass correlation; CI; Confidence Interval; SD, Standard Deviation; SEM, Standard Error of Measurement; *number of participants = 19 N; Newtons

*Temporal summation*. Table 6 summarises all ICCs for M1, M2, M3 and ratios (M3/M1). A number of participants asked for temporal summation testing to stop due to high pain levels therefore a full set of NRS were not collected. Where a full set of 10 NRS were not collected, these were excluded from the analyses with Table 6 summarising number of participants included in analyses. For intra-rater reliability, ICCs for the three means (M1, M2, M3) ranged from poor to excellent (ICC range 0.20, 0.91) with the lower limb sites overall showing a wider range in ICCs compared to other sites. For inter-rater reliability, day one and day two ICCs for the means were all rated poor to excellent (ICC range 0.30, 0.92). For the ratio ICCs both intra and inter-rater were rated as poor or moderate (ICC range -0.31, 0.66) with the exception of right upper limb site which was rated as good (ICC 0.86).

**Table 6:** Intraclass correlations and means for temporal summation for the asymptomatic participants.

Site		Mean (SD) Day 1		Mean (SD) Day 2		ICC (95% CI) Intra-Rater		ICC (95% CI) Inter-Rater		ICC (95% (CI) Ratio Intra-Rater		ICC (95% CI) Ratio Inter-rater	
		Rater 1	Rater 2	Rater 1	Rater 2	Rater 1	Rater 2	Day 1	Day 2	Rater 1	Rater 2	Day 1	Day 2
ULR	M1	4.27 (1.97)	4.48 (1.77)	3.98 (1.77)	4.31 (1.61)	0.60 (0.04, 0.87)	0.74 (0.31, 0.91)	0.73 (0.28, 0.90)	0.71 (0.27, 0.88)	-0.31 (-2.67,0.52)	0.32 (-0.94, 0.75)	0.82 (0.53, 0.93)	-0.02 (-1.33, 0.58)
	M2	5.04 (2.17)	5.17 (2.06)	4.78 (1.78)	4.95 (1.75)	0.62 (-0.02, 0.86)	0.70 (0.20, 0.89)	0.72 (0.22, 0.90)	0.72 (0.29, 0.89)				
	M3	5.14 (2.24)	5.40 (2.18)	5.18 (2.07)	4.98 (1.88)	0.82 (0.52, 0.93)	0.78 (0.42, 0.92)	0.80 (0.46, 0.92)	0.77 (0.41, 0.91)				
ULL	M1	4.62 (2.21)	4.52 (1.99)	3.77 (1.54)	4.58 (1.89)	0.79 (0.39, 0.93)	0.91 (0.73, 0.97)	0.77 (0.39, 0.91)	0.83 (0.40, 0.95)	0.55 (-0.22, 0.84)	0.56 (-0.27, 0.85)	0.66 (0.15, 0.86)	0.86 (0.59, 0.95)
	M2	5.07 (2.40)	5.28 (2.10)	4.48 (2.00)	5.04 (1.87)	0.65 (-0.06, 0.88)	0.87 (0.63, 0.96)	0.68 (0.14, 0.88)	0.76 (0.33, 0.91)				
	M3	5.10 (2.74)	5.60 (2.23)	3.52 (2.11)	5.06 (1.98)	0.77 (0.31, 0.93)	0.87 (0.62, 0.96)	0.65 (0.10, 0.87)	0.89 (0.69, 0.96)				
LLR	M1	3.96 (1.93)	3.81 (1.65)	3.64 (1.37)	3.59 (1.20)	0.58 (-0.10, 0.84)	0.77 (0.40, 0.91)	0.73 (0.29, 0.90)	0.30 (-0.87, 0.73)	-0.04 (-1.73, 0.60)	0.23 (-1.09, 0.71)	-0.17 (-2.23, 0.56)	0.38 (-0.66, 0.76)
	M2	4.54 (2.23)	4.49 (2.16)	4.55 (1.56)	4.37 (1.43)	0.23 (-1.1, 0.71)	0.83 (0.56, 0.94)	0.77 (0.39, 0.91)	0.35 (-0.71, 0.75)				
	M3	4.78 (2.30)	5.08 (2.39)	4.63 (1.88)	4.60 (1.95)	0.20 (-1.18, 0.70)	0.82 (0.53, 0.93)	0.84 (0.58, 0.94)	0.56 (-0.11, 0.83)				
LLL	M1	3.95 (1.82)	3.86 (1.76)	4.19 (1.74)	4.05 (1.67)	0.88 (0.71, 0.95)	0.63 (0.04, 0.85)	0.76 (0.39, 0.91)	0.74 (0.33, 0.90)	0.27 (-0.69, 0.70)	0.41 (-0.26, 0.75)	0.35 (-0.48, 0.73)	0.46 (-0.18, 0.77)
	M2	4.60 (2.10)	4.57 (1.93)	5.15 (1.94)	4.45 (1.95)	0.70 (0.26, 0.88)	0.66 (0.13, 0.87)	0.75 (0.36, 0.90)	0.70 (0.27, 0.88)				
	M3	4.63 (2.31)	5.15 (2.08)	5.43 (2.01)	4.60 (1.87)	0.61 (0.07, 0.84)	0.63 (0.10, 0.85)	0.84 (0.61, 0.94)	0.63 (0.12, 0.85)				
SR	M1	3.61 (1.50)	3.60 (1.77)	3.59 (1.71)	3.69 (1.48)	0.75 (0.35, 0.91)	0.58 (-0.16, 0.85)	0.69 (0.19, 0.88)	0.84 (0.57, 0.94)	0.61 (-0.09, 0.86)	0.01 (-1.72, 0.63)	0.01 (-1.59, 0.62)	0.62 (-0.03, 0.85)
	M2	3.78 (1.95)	3.82 (1.90)	3.93 (1.82)	3.93 (1.56)	0.51 (-0.35, 0.82)	0.81 (0.48, 0.93)	0.72 (0.26, 0.89)	0.71 (0.23, 0.89)				
	M3	4.19 (2.19)	4.05 (2.08)	3.96 (1.82)	4.12 (1.73)	0.61 (0.00, 0.85)	0.76 (0.35, 0.91)	0.70 (0.20, 0.88)	0.77 (0.40, 0.91)				
SL	M1	3.65 (1.77)	3.76 (1.77)	3.50 (1.80)	3.83 (1.80)	0.90 (0.75, 0.96)	0.81 (0.50, 0.23)	0.56 (-0.14, 0.83)	0.86 (0.65, 0.94)	0.10 (-1.44, 0.65)	0.89 (0.72, 0.96)	-0.03 (-1.63, 0.59)	0.26 (-0.85, 0.71)
	M2	4.17 (2.10)	4.32 (1.67)	3.89 (1.99)	4.27 (2.11)	0.84 (0.61, 0.94)	0.78 (0.42, 0.91)	0.49 (-0.34, 0.80)	0.92 (0.80, 0.97)				
	M3	4.40 (2.43)	4.45 (1.86)	4.00 (2.15)	4.50 (2.25)	0.83 (0.57, 0.93)	0.73 (0.31, 0.89)	0.59 (-0.07, 0.84)	0.92 (0.78, 0.97)				

ULR, Upper Limb Right; ULL, Upper Limb Left; LLR, Lower Limb Right; LLL, Lower Limb Left; SR, Spine Right; SL, Spine Left; ICC, Intraclass correlation; CI; Confidence Interval; SD, Standard Deviation; SEM, Standard Error of Measurement

Intra-Rater Number of Participants ULL = 15, ULR/SR = 18, LLR = 19, LLL/SR = 20 Inter-Rater Day 1 Number of Participants ULR = 18, ULL/LLR/SR = 19, LLL/SL = 20

Inter-Rater Day 2 Number of Participants ULL = 16, SR = 19, ULR/LLR/LLL/SL = 20

#### Acute musculoskeletal trauma participants

*Heat pain thresholds*. A total of three sets of data for the remote site were excluded from analyses due to pain not being evoked within the safety limit of the thermal testing device (50.5^0^). No sets of data were excluded for the local site analyses. Both local (ICC 0.86 CI 0.65, 0.95) and remote (ICC 0.81 CI 0.49, 0.93) sites were rated as good for HPT. For SEM, rater 1 for the local site was 1.02°C and less for the remote site at 0.85°C. For rater 3, both sites were similar to rater 1 with the local site SEM being 1.11°C, and remote site 1.24°C. ICCs with 95% CIs, SEM and means for both local and remote sites for HPT are reported in Table 7.

**Table 7:** Intraclass correlations, means and standard error of measurement for heat and cold pain thresholds, pressure pain thresholds and temporal summation for the acute musculoskeletal trauma participants.

Site	Modality	Number of participants included in analyses	Rater 1 Mean (SD)	Rater 3 Mean (SD)	ICC (95% CI)	Standard Error of Measurement	
						Rater 1	Rater 3
Local Site	HPT ^0^C	N = 20	46.92 (2.72)	46.59 (2.97)	0.86 (0.65, 0.95)	1.02	1.11
	CPT ^0^C	N = 7	13.53 (8.87)	12.90 (6.80)	N/A	N/A	N/A
	PPT Newtons	N = 20	37.60 (12.87)	36.26 (12.94)	0.78 (0.43, 0.91)	6.04	6.07
	Mean 1	N = 17	5.25 (1.88)	5.35 (1.79)	0.87 (0.63, 0.95)	N/A	N/A
	Mean 2	N = 17	5.16 (1.49)	5.10 (2.03)	0.69 (0.13, 0.89)	N/A	N/A
	Mean 3	N = 17	5.59 (1.75)	5.00 (2.14)	0.79 (0.45, 0.92)	N/A	N/A
	Ratio between Mean 3 and Mean 1 (NRS)	N = 17	1.15 (0.46)	0.96 (0.31)	0.57 (-0.07, 0.84)	N/A	N/A
Remote Site	HPT ^0^C	N = 17	47.75 (1.95)	47.06 (2.85)	0.81 (0.49, 0.93)	0.85	1.24
	CPT ^0^C	N = 3	11.84 (5.57)	7.37 (1.46)	N/A	N/A	N/A
	PPT Newtons	N = 20	43.36 (14.18)	42.22 (15.20)	0.76 (0.43, 0.91)	6.95	7.45
	Mean 1 (NRS)	N = 18	4.96 (2.28)	5.35 (2.47)	0.91 (0.77, 0.97)	N/A	N/A
	Mean 2 (NRS)	N = 18	5.38 (2.46)	5.75 (2.54)	0.94 (0.84, 0.98)	N/A	N/A
	Mean 3 (NRS)	N = 18	5.47 (2.62)	5.90 (2.51)	0.93 (0.82, 0.97)	N/A	N/A
	Ratio between Mean 3 and Mean 1 (NRS)	N = 18	1.11 (0.31)	1.13 (0.32)	0.62 (-0.04, 0.86)	N/A	N/A

HPT, heat pain threshold; CPT, cold pain threshold; PPT, pressure pain threshold; NRS, numerical rating scale; SD, standard deviation; ICC, Intraclass correlation coefficient

*Cold pain thresholds*. Thirteen participants were excluded from cold data analyses as pain was not evoked within the safety limit of the thermal testing equipment for the local site and 17 participants excluded for the remote site. Therefore, a total of 7 participants for the local site and 3 for the remote site were included in analyses. Due to low numbers of participants included in data analyses, descriptive data only are reported. A summary of the descriptive data for CPT can be found in Table 7.

*Pressure pain thresholds*. Both local (ICC 0.78 CI 0.43, 0.91) and remote (ICC 0.76 CI 0.43, 0.91) were rated as good. The SEM for rater 1was 6.04N for the local site and 6.95N for the remote site. For rater 3, SEM was similar to rater 1 with the 6.07N for the local site and 7.45N for the remote site. ICCs with 95% CIs, SEM and means for both local and remote sites for PPT are reported in Table 7.

*Temporal summation*. Three sets of data were excluded from analyses for the local site and two sets of data for the remote site due to the participant asking for testing to stop due to high pain levels therefore 10 pulses were not achieved. When calculating the ratio both local (ICC 0.57 CI -0.07, 0.84) and remote (ICC 0.62 CI -0.04, 0.86) were rated as moderate. However, when calculating the 3 means over the 10 pulses, for the local site M1 (ICC 0.87 CI 0.63, 0.95) and M3 (ICC 0.79 CI 0.45, 0.92) were rated as good whereas M2 (ICC 0.69 CI 0.13, 0.89) was rated as moderate. For the remote site all three means were rated as excellent (M1 ICC 0.91 CI 0.77, 0.97, M2 ICC 0.94 CI 0.84, 0.98, M3 ICC 0.93 CI 0.82, 0.97). ICCs with 95% CIs and means for both local and remote sites for TS are reported in Table 7.

#### Bland Altman

**Fig 1:**
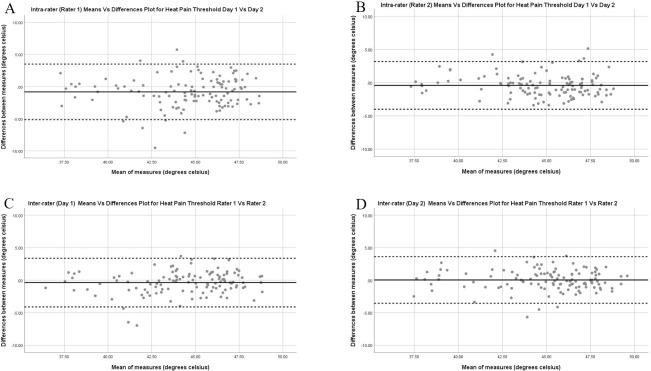
Intra-rater (A & B) and Inter-rater (C & D) Bland Altman Plots with limits of agreement for heat pain thresholds for the asymptomatic participants. Limits of agreement are presented as the dotted lines with the mean difference illustrated by the black line.

**Fig 2:**
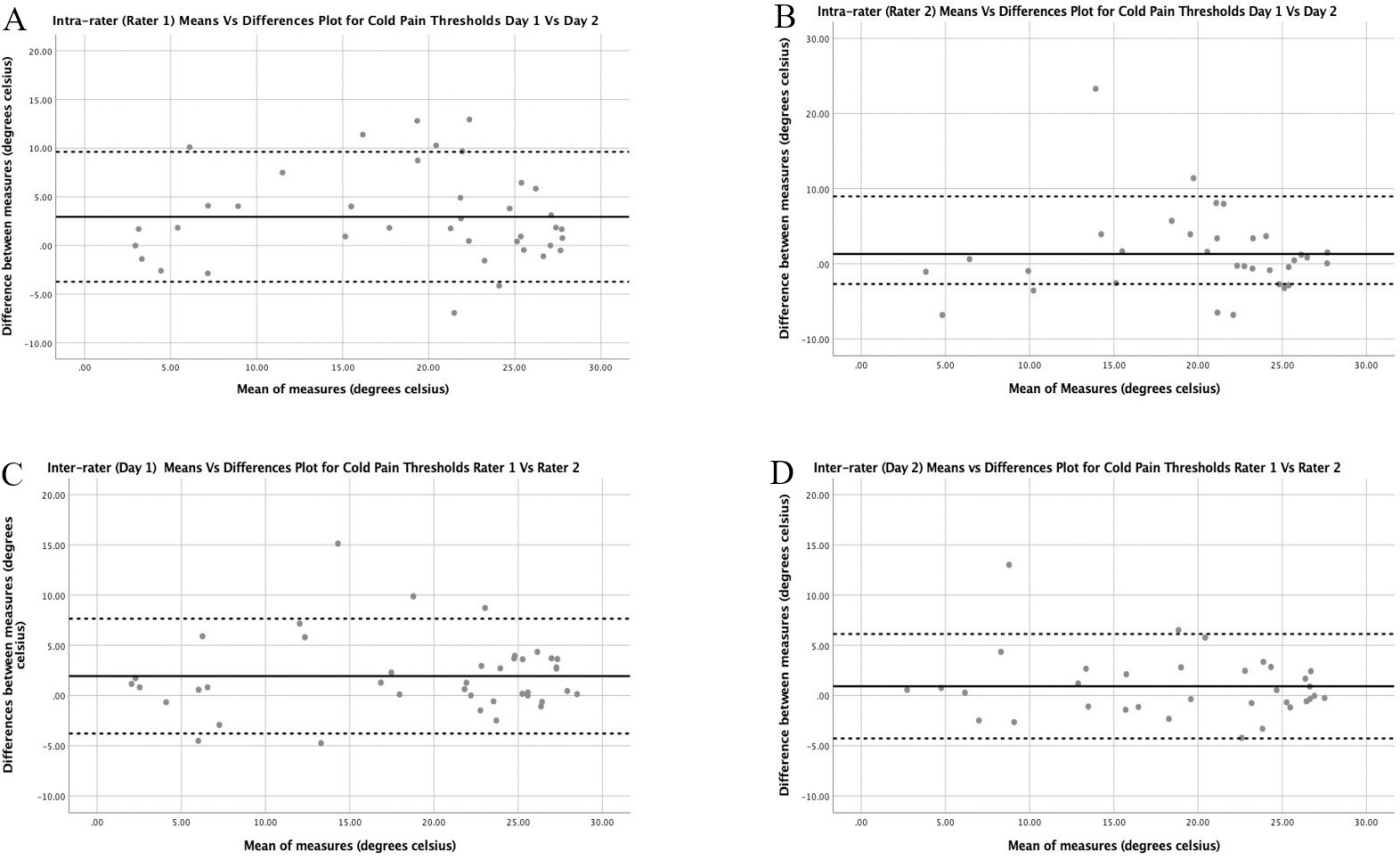
Intra-rater (A & B) and Inter-rater (C & D) Bland Altman Plots with limits of agreement for cold pain thresholds for the asymptomatic participants. Limits of agreement are presented as the dotted lines with the mean difference illustrated by the black line.

**Fig 3:**
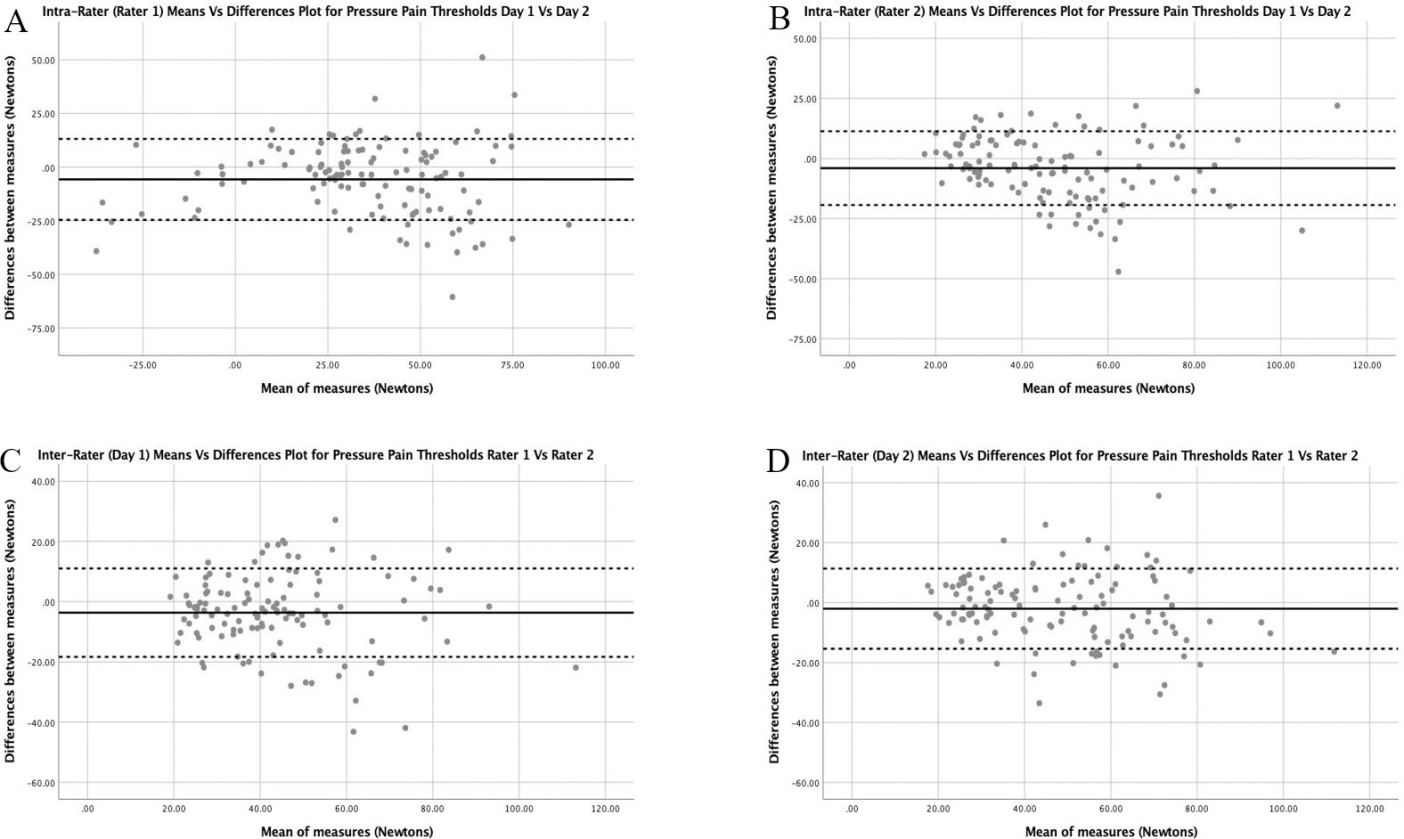
Intra-rater (A & B) and Inter-rater (C & D) Bland Altman Plots with limits of agreement for pressure pain thresholds for the asymptomatic participants. Limits of agreement are presented as the dotted lines with the mean difference illustrated by the black line.

*Heat pain thresholds*. Figs 1–3 depict the Bland Altman plots and limits of agreement for HPT, CPT and PPT for the asymptomatic participants for both intra and inter-rater reliability. The Bland Altman plots for HPT (Fig 1) show similar limits of agreement with the average mean difference close to 0°C with no systematic or proportional bias demonstrated.

The Bland Altman plots for CPT (Fig 2) show more variation in limits of agreement with Rater 1 intra-rater average mean difference at 2.95°C whereas rater 2 mean difference at 1.30°C. Inter-rater agreement between raters was improved on day 2 compared to 1. Although more variation in agreement, no systematic or proportional bias was demonstrated.

The Bland Altman plots for PPT (Fig 3) show similar limits of agreement for both raters and both days. For both intra and inter rater agreement, less agreement appears to be consistent in higher pain thresholds compared to lower pain thresholds, however no systematic or proportional bias is evident.

**Fig 4:**
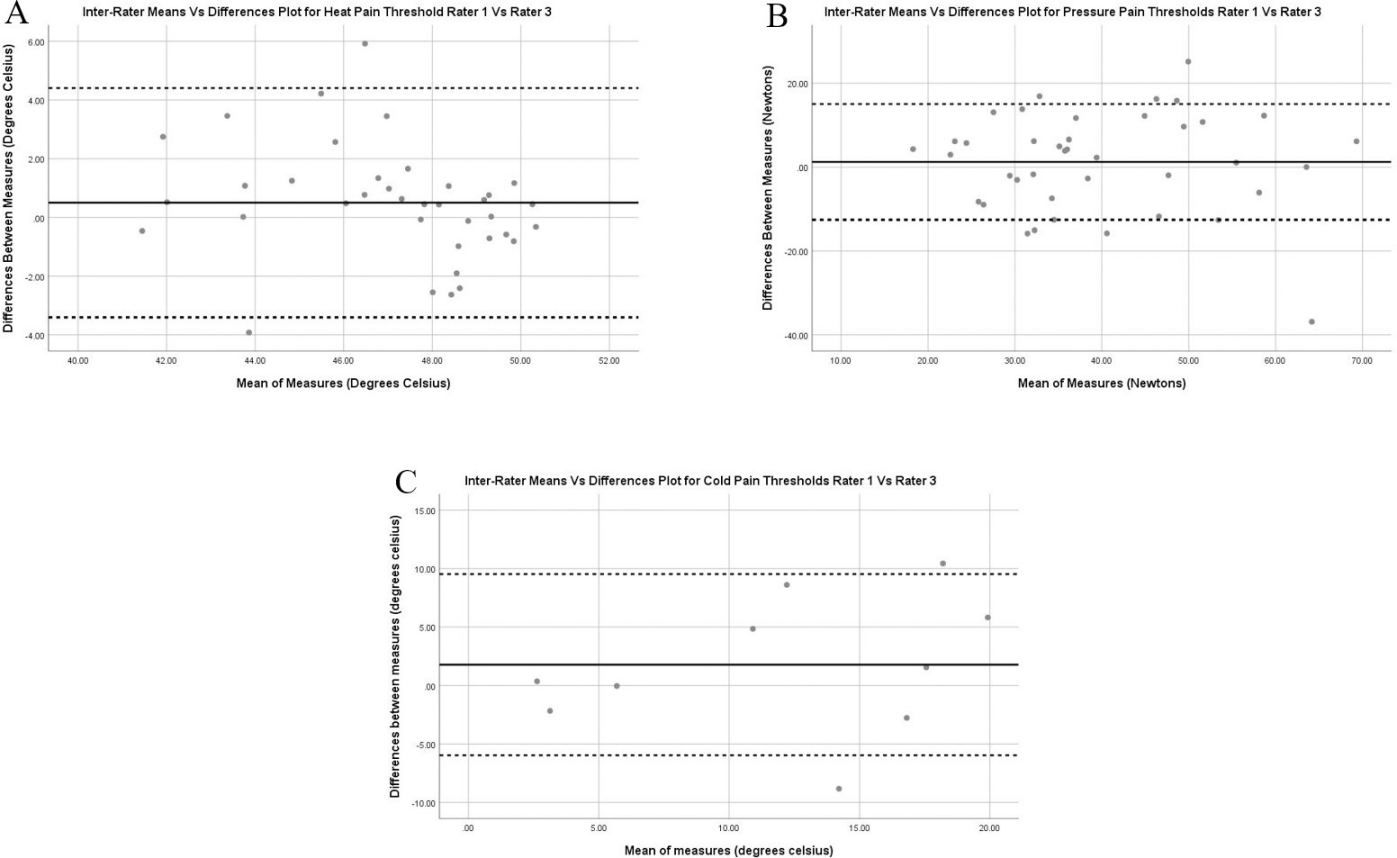
Inter-rater Bland Altman Plots with limits of agreement for heat pain thresholds (A), pressure pain thresholds (B) and cold pain thresholds (C) for the acute musculoskeletal trauma participants. Limits of agreement are presented as the dotted lines with mean difference the black line.

Fig 4 depicts the Bland Altman plots and limits of agreement for HPT, CPT and PPT for the acute musculoskeletal trauma participants. All plots show good agreement between raters with the average mean difference no more than 1°C or 1N. For HPT, higher temperature scores are associated with rater 3 compared to rater 1, and further investigation into the dataset showed this to be more for the remote site than local site. No other proportional or systematic bias was shown in for PPT and CPT.

## Discussion

This is the first methodologically rigorous reliability study evaluating intra and inter-rater reliability within asymptomatic participants and inter-rater reliability for acute musculoskeletal trauma participants. In asymptomatic participants, reliability was established as moderate to good intra-rater reliability for HPT and PPT, with the exception of one lower limb site for PPT which was rated poor, and moderate to excellent inter-rater reliability for PPT and HPT. Good to excellent inter-rater reliability of HPT and PPT was demonstrated for acute musculoskeletal trauma participants. Bland Altman plots supported these findings with good agreement between days and raters for HPT and PPT, however agreement for PPT was less with higher PPT scores (i.e. more pressure exerted by the rater) compared to lower scores (less pressure exerted by the rater) more so in the asymptomatic participants. SEM for both HPT and PPT was low, with PPT SEM in asymptomatic participants having more variability depending on the testing site. TS demonstrated variable results ranging from poor to excellent reliability in asymptomatic participants for intra and inter-rater reliability, but moderate to excellent reliability for acute musculoskeletal trauma participants. However, some participants in both groups asked for testing to stop due to high pain intensity during TS. Limited conclusions can be made for CPT since no statistical analyses could be performed yet, Bland Altman plots show adequate agreement in both sets of participants.

No previous study has evaluated the reliability of these specific QST tests in combination, however, results in this study are consistent with studies evaluating thermal and pressure testing as stand-alone modalities, [19, 22, 26, 52, 53] as well as recent studies combining multiple QST tests [27] and studies using the established DNFS protocol for QST [24, 25].

Although the current study demonstrated good reliability for PPT in both sets of participants, musculoskeletal trauma participants showed higher inter-rater reliability compared to asymptomatic participants. This was also observed for TS testing, where results for asymptomatic participants were more variable compared to the musculoskeletal trauma participants. A possible reason for the variation compared to previous studies could be due to the different algometers used across studies including a digital algometer [29, 53] or a mechanical algometer [19]. The algometer used in the current study was a digital handheld algometer, with an additional software programme used to guide rate of pressure and to accurately calculate the temporal summation rate for each participant. Because of this, raters were required to focus on the computer screen and not on the site during testing, which could have possibly led to variation in the technique applied, although both raters had substantial training on the method prior to commencement of the study. This could also explain why TS reliability results were variable given that same method of measuring PPT was used for TS testing.

This algometer however, was used in both sets of participants and does not explain differences in results between the asymptomatic and acute musculoskeletal trauma participants. For the asymptomatic participants, the testing protocol was extensive using six sites with four modalities which totals to 24 tests all focusing on pain thresholds. It is possible the quantity of testing caused habituation or sensitisation to testing thus having the potential for their pain thresholds to increase or decrease between sessions [54–56]. This could have the potential to explain the variability in TS results which was not observed for musculoskeletal trauma participants. Research in this area is limited, with a handful of studies evaluating the habituation/sensitisation effect [54–56]. These studies however focus on thermal testing only, over a number of days with testing every day, which differs from the multiple modalities and testing schedule used in this study. However, other protocols such as the DNFS protocol have extensive QST testing [57]. Although the DNFS protocol is extensive, all tests are not pain threshold tests but combine detection thresholds differing to the protocol for this study. Future research is required in this area to evaluate the effects of QST and the effects of habituation and sensitisation. Despite this, future studies should also ensure training of raters prior to testing, as this study has highlighted good inter-rater reliability can be achieved both in asymptomatic and acute musculoskeletal trauma participants.

Despite the differences in PPT and TS, HPT was consistently rated good or excellent for both asymptomatic and acute musculoskeletal trauma participants, with SEM for HPT being approximately 1°C for both sets of participants. However, across all modalities a trend was observed whereby both SEM and ICCs on day 2 of testing of the asymptomatic participants increased. This could indicate a potential learning effect has taken place. The raters received training before undertaking the study and since no systematic or proportional bias is demonstrated in the Bland Altman plots for the asymptomatic participants, it is unlikely there is a learning effect from the raters which could contribute to the variation in results, however despite a period of test familiarisation for the participants prior to testing, a learning effect could have taken place which could explain increased reliability on day 2. Furthermore, a specific testing protocol was adopted for this study with a minimum of 2 hours between sessions observed to allow a period of rest for the participant and to minimise any potential bias and learning effect, with 48 hours given between testing days for the asymptomatic participants. Previous studies have used a range of times from five minutes to ten minutes for within day testing between different raters [19, 29] and up to four months for between day testing for intra-rater reliability [24]. With no consensus on the amount of time between sessions, the larger time period between sessions and days of testing in this study may have contributed to differences between days of testing. Future studies should take into consideration a potential learning effect and consider a familiarisation session on a different day rather than immediately before the testing to account for this. No short-term learning effect was observed or indicated for within day testing for the asymptomatic or acute musculoskeletal trauma participants.

Taking into account the results of the reliability studies conducted, and the confounding factors which can influence the reliability of measures such as time between testing sessions, number of sites tested, adequate training of raters and the position and methods of the algometer for PPT and TS testing, this study sets the foundations for future studies to utilise QST measures within clinical research. This can include investigating if early evidence of sensitisation relates to how well people recover from injury. However, it is important for studies to acknowledge the potential factors which can influence results of QST testing and factor this into study design to ensure a change in pain thresholds is captured rather than measurement error.

### Strengths and limitations

This is the first study to evaluate intra and inter-reliability of CPT, HPT, PPT and TS combined, and the first study to evaluate inter-rater reliability in an acute hospital setting for a musculoskeletal trauma population. Establishing reliability for these measures allows future studies to evaluate sensory changes from an acute stage of injury over time as people recover from traumatic musculoskeletal injuries and further demonstrate the value of QST in predicting outcome [40]. Additionally, previous reliability studies have controlled the environment in which testing was conducted, often within a lab setting. Although the asymptomatic sample was conducted within the lab, the environment was not controlled as strictly as previous studies where temperature and background noise were controlled. This was deliberate, as there is a considerable amount of noise, distraction and variance in temperature within a hospital setting, and reliability of these measures needed to be established in these real-life clinical conditions in order to be transferable to future studies.

Nevertheless, there are some limitations to this study. One limitation to this study is we had low participant numbers for CPT due to the safety limit of the thermal analyzer, making overall conclusions to the reliability of CPT difficult. One study has acknowledged similar issues with participants reaching the safety limit data [22] and excluded this data from analysis. The decision was made to exclude this data in the current study, as if included, the ICCs would likely portray excellent reliability and agreement, which could be misleading. Although conclusions of the reliability of CPT cannot be made from this study, the reporting of the issues with the safety limit of the thermal analyzer is important to document. Even though the device was calibrated correctly and the application followed protocols described in previous studies, issues were still observed. This has implications for future studies using CPT as well as future studies required to establish reliability of CPT with adequate sample sizes.

Secondly, due to high pain levels experienced in some participants for TS testing, sample sizes for some sites were lower than 19. Therefore, some ICC’s were slightly underpowered but had adequate numbers to perform statistical analysis [37, 38]. This should be taken into consideration when interpreting the results of the TS testing. Although we are less confident in the ICC reported, it does not change the overall conclusions of this study that the TS protocol requires further testing and evaluation.

Another limitation of this study was inter-rater reliability but not intra-rater reliability was evaluated within the musculoskeletal trauma population. Due to the acute nature of musculoskeletal trauma, assessing reliability over a number of days was difficult, as symptoms and pain are often changeable making it problematic to assess reliability rather than change of symptoms. Although intra-rater could have been tested on the same day, the decision was made to evaluate inter-rater reliability, as intra-rater reliability in the asymptomatic participants was moderate to good depending on modality, and inter-rater reliability is clinically relevant for future studies planned [40].

Finally, although this study has demonstrated good reliability for the battery of QST tests used, the ICC statistical model (two-way mixed effects, absolute agreement 3,2) was chosen as the particular raters used in this study were of interest, therefore the more common ICC statistical model of two-way random effects utilised when the raters of interest are selected from random and therefore can be generalised to a wider population of raters with similar characteristics was not appropriate for this study [50]. Therefore, generalising the study to larger populations and other raters should be interpreted with caution and further research is required to confirm these results.

## Conclusion

This study has demonstrated moderate to good intra-rater reliability and moderate to excellent inter-rater reliability for HPT and PPT in a sample of asymptomatic individuals with good to excellent inter-rater reliability demonstrated for HPT and PPT in an acute musculoskeletal trauma population. Good reliability was demonstrated for TS within the acute musculoskeletal trauma population. Limited conclusions can be made of CPT data, however Bland Altman plots did show good agreement between days and raters. This study has demonstrated HPT, CPT, PPT and TS can be used in an acute clinical environment and forms the foundation for future work in acute musculoskeletal trauma population evaluating sensory function over time as well as the value of QST in predicting outcome post trauma.
